# Deciphering the immune-metabolic nexus in sepsis: a single-cell sequencing analysis of neutrophil heterogeneity and risk stratification

**DOI:** 10.3389/fimmu.2024.1398719

**Published:** 2024-07-23

**Authors:** Shaoxiong Jin, Huazhi Zhang, Qingjiang Lin, Jinfeng Yang, Rongyao Zeng, Zebo Xu, Wendong Sun

**Affiliations:** ^1^ Department of Emergency Surgery, The Second Affiliated Hospital of Fujian Medical University, Quanzhou, Fujian, China; ^2^ Department of Radiology, The Second Affiliated Hospital of Fujian Medical University, Quanzhou, Fujian, China

**Keywords:** sepsis, single-cell sequencing, neutrophils, metabolic dysregulation, risk score model

## Abstract

**Background:**

Metabolic dysregulation following sepsis can significantly compromise patient prognosis by altering immune-inflammatory responses. Despite its clinical relevance, the exact mechanisms of this perturbation are not yet fully understood.

**Methods:**

Single-cell RNA sequencing (scRNA-seq) was utilized to map the immune cell landscape and its association with metabolic pathways during sepsis. This study employed cell-cell interaction and phenotype profiling from scRNA-seq data, along with pseudotime trajectory analysis, to investigate neutrophil differentiation and heterogeneity. By integrating scRNA-seq with Weighted Gene Co-expression Network Analysis (WGCNA) and machine learning techniques, key genes were identified. These genes were used to develop and validate a risk score model and nomogram, with their efficacy confirmed through Receiver Operating Characteristic (ROC) curve analysis. The model’s practicality was further reinforced through enrichment and immune characteristic studies based on the risk score and *in vivo* validation of a critical gene associated with sepsis.

**Results:**

The complex immune landscape and neutrophil roles in metabolic disturbances during sepsis were elucidated by our in-depth scRNA-seq analysis. Pronounced neutrophil interactions with diverse cell types were revealed in the analysis of intercellular communication, highlighting pathways that differentiate between proximal and core regions within atherosclerotic plaques. Insight into the evolution of neutrophil subpopulations and their differentiation within the plaque milieu was provided by pseudotime trajectory mappings. Diagnostic markers were identified with the assistance of machine learning, resulting in the discovery of PIM1, HIST1H1C, and IGSF6. The identification of these markers culminated in the development of the risk score model, which demonstrated remarkable precision in sepsis prognosis. The model’s capability to categorize patient profiles based on immune characteristics was confirmed, particularly in identifying individuals at high risk with suppressed immune cell activity and inflammatory responses. The role of PIM1 in modulating the immune-inflammatory response during sepsis was further confirmed through experimental validation, suggesting its potential as a therapeutic target.

**Conclusion:**

The understanding of sepsis immunopathology is improved by this research, and new avenues are opened for novel prognostic and therapeutic approaches.

## Introduction

Sepsis is a life-threatening condition characterized by a dysregulated host response to infection, which can lead to organ dysfunction. Globally, sepsis is estimated to affect over 30 million people annually, potentially resulting in 6 million deaths (1). Currently, the management of sepsis relies on prompt antibiotic therapy, removal of the infection source, and supportive measures to maintain hemodynamic stability and preserve organ function (2). However, patient-specific response variability complicates management, reflecting the limited understanding of sepsis pathogenesis and signaling the need for more effective, individualized treatment approaches. Tailored therapies, founded on patient-specific biomarkers and stratification based on immunological or genetic profiles, can enhance effectiveness and reduce the likelihood of adverse effects.

Sepsis initiates a dynamic immune response that evolves over time, marked by concurrent pro-inflammatory and anti-inflammatory processes. Consequently, most sepsis patients rapidly exhibit signs of profound immune suppression, resulting in detrimental outcomes (3). Recent research has emphasized the significance of metabolic dysfunction, epigenetic changes, myeloid-derived suppressor cells, immature neutrophils with suppressive properties, and immune variations in main lymphoid organs during sepsis (4–6).

Metabolic dysfunction plays a crucial role in the initiation and progression of sepsis. During sepsis, the body undergoes an advanced level of metabolic emergency which can potentially lead to organ dysfunction (7). Metabolic shifts are prominently observed in various cell types during sepsis, particularly as immune cells undergo transformation. Cellular metabolism, which exhibits variable metabolic profiles across different cell types and stages of the disease, plays a key role in the immune dysregulation and organ failure associated with sepsis (3, 8). Metabolic reprogramming, wherein glycolysis supersedes oxidative phosphorylation (OXPHOS) as the primary source of energy production, is crucial for immune cell activation while simultaneously contributing to immunosuppression (9). Additionally, metabolites from OXPHOS and glycolysis may serve as signaling molecules, modulating the immune response throughout sepsis. The “energy crisis” induced by sepsis leads to impaired cellular functions and potentially severe organ dysfunction (10). Although metabolic reprogramming can partially mitigate this energy deficit, fostering host tolerance and enhancing cell survival, reversion to OXPHOS is imperative for cellular function restoration (11). In the intricate landscape of molecular and cellular biology, significant rewiring of metabolic pathways and epigenetic modifications has been identified as a pivotal factor in triggering and perpetuating immune system changes linked to sepsis. These alterations precipitate profound changes in gene expression patterns which lie at the heart of sepsis-induced immunological transformations (12). From a broader perspective, immune cells require metabolic profile alterations to achieve effective functionality. These metabolic changes are tentatively linked to the progression of immune responses during sepsis (13). This metabolic deceleration is akin to the cellular hibernation noted in organ dysfunction related to sepsis (14). Therefore, exploring the interplay between metabolism and immunity in the context of sepsis is a critical area of research, pivotal for identifying novel therapeutic targets to restore immune homeostasis following sepsis.

In this study, scRNA-seq was employed to investigate the immune cell composition of sepsis patients, revealing a specific enrichment of immune cell types. The application of the MuSiC algorithm and intercellular communication assessments uncovered notable interactions among immune cells, highlighting the crucial role of neutrophils in sepsis and their connection to metabolic activity. The analysis of neutrophil heterogeneity has led to the identification of four distinct subtypes, each characterized by unique functional attributes. Furthermore, the developmental trajectories of neutrophils were traced, leading to the identification of essential genes and the characterization of subpopulation lineage differentiation. By utilizing WGCNA, gene co-expression networks were constructed to identify significant genes for further investigation. Gene enrichment assays were then performed to elucidate the biological functions of these genes. Machine learning algorithms were employed to identify potential biomarkers for atherosclerosis, leading to the development of a diagnostic model with enhanced predictive capabilities. Using a customized riskScore model, we stratified patients based on their risk profiles and investigated the molecular and immune characteristics associated with different risk levels. The validation of characteristic genes *in vivo* sepsis models underscored the significance of these targeted genes in the disease’s pathology.

## Methods

### Acquisition of raw data

scRNA-seq data of peripheral blood from a cohort of five healthy individuals and four patients with advanced-stage sepsis were retrieved from the Gene Expression Omnibus (GEO) repository, specifically under accession number GSE175453. Concurrently, aggregated transcriptomic datasets associated with sepsis were acquired from GEO (accession numbers: GSE65682, GSE95233, GSE63042) and the ArrayExpress database (accession number: E-MTAB-5273). After the acquisition, these datasets underwent a logarithmic transformation to base 2 and normalization utilizing the Robust Multi-array Average (RMA) algorithm available within the “affy” package in the R statistical environment.

### scRNA-seq data processing and cell annotation

Utilizing the R package “Seurat,” single-cell RNA sequencing (scRNA-seq) data was analyzed with meticulous attention to precision. Initially, the dataset underwent a rigorous gene filtering process where only genes present in no fewer than three individual cells were considered for further examination. This initial step ensures that the focus remains on genes with sufficient representation across the cell population, thus enhancing the robustness of downstream analyses. Concomitantly, cells were filtered based on their gene expression profiles, retaining cells that exhibited an expression range of 200 to 3000 genes. This specific criterion was set to exclude cells with abnormally low or high gene counts, which could otherwise introduce noise into the dataset.

Additionally, cells were subjected to further filtering based on two additional parameters: the total RNA count (nCount_RNA) and mitochondrial gene expression. Specifically, a threshold was established to retain cells with an nCount_RNA below 20,000 to exclude potential doublets or multiplets that could distort the results. Mitochondrial gene expression was also monitored and kept under 10%, as an elevated mitochondrial gene percentage is often indicative of low-quality or dying cells. These stringent filtering criteria ensured that only high-quality cells were retained, culminating in a dataset comprising 40,584 cells deemed suitable for advanced analyses.

To prepare the selected cell population for subsequent steps, the data was normalized and scaled using Seurat’s “NormalizeData” and “ScaleData” functions. Normalization adjusted the gene expression measurements for each cell to account for differences in sequencing depth, resulting in the expression levels on a comparable scale across all cells. Scaling further refined these measurements by centering the data and scaling each gene to unit variance, thereby mitigating the effects of any highly variable or abundant genes.

Following this preliminary processing, the most variable genes were identified to capture the underlying biological heterogeneity within the cell population. Using the “FindVariableFeatures” function in Seurat, the top 3,000 genes exhibiting the highest variability across the dataset were pinpointed. Given the dataset’s multi-sample origin, it was crucial to address potential batch effects that could confound the analyses. This was achieved using the “RunHarmony” function, which harmonizes the data across different samples, thereby reducing batch-induced biases.

Subsequent dimensionality reduction was performed using principal component analysis (PCA), a technique that enables the condensation of the data’s complexity by projecting it into a set of orthogonal components. We focused on the top 20 principal components, which encapsulated the most significant variance in the dataset. To further elucidate cell population structures, these components were subjected to t-distributed stochastic neighbor embedding (t-SNE) analysis, which projected the high-dimensional data into a two-dimensional space. This visualization technique facilitated the discernment of significant cellular conglomerates and patterns.

In the clustering phase, Seurat’s “FindNeighbors” and “FindClusters” functions were executed, with the latter set to a resolution parameter of 0.3. This clustering approach partitioned the cells into 13 distinct clusters. The resolution parameter was tuned to balance the granularity of the clusters, ensuring a meaningful yet interpretable clustering outcome. The resulting clusters were visually represented in a t-SNE plot, providing an intuitive overview of the cellular landscape.

Subsequent cluster annotation involved a thorough manual examination, wherein each cluster was classified into major cell types based on established marker gene profiles. Marker genes serve as distinctive identifiers for various cell types, allowing for accurate classification. To characterize the markers within each cellular contingent, the “COSG” package in R was employed. The parameters were specifically configured with a mean expression threshold of 10 and a user-defined gene count of 100, facilitating a comprehensive and precise marker characterization essential for downstream biological interpretations.

### Evaluation of metabolic activity at single-cell resolution

In each cell population, the metabolic processes of singular cells were mapped and measured utilizing the ‘scMetabolism’ package in R, a cutting-edge tool designed for single-cell metabolic activity quantification (15). This tool processes a matrix of single-cell data, employing the VISION algorithm to assess individual cell metabolic pathway scores. Embedded within the ‘scMetabolism’ tool are the comprehensive KEGG and Reactome pathway databases. Before the metabolic examination, the dataset underwent a uniform transformation. The VISION algorithm played a pivotal role in computing the metabolic scores. Comparative analysis of metabolic activities across different cellular groupings pinpointed pathways with notable variances. For this investigation, the analysis harnessed KEGG metabolic gene sets coupled with the “VISION” technique. Subsequently, for graphical representation, we utilized the “DotPlot.metabolism” and “BoxPlot.metabolism” functions.

### Annotating cell types in bulk RNA−seq dataset

Single Cell Multi-Subject (SCMS) serves as an effective approach to determining the prevalence of distinct cell populations. This methodology applies gene expression profiles particular to each cell type obtained from scRNA-seq to ascertain the comparative frequency of a range of cell subsets within a composite RNA-seq dataset. To appraise the respective contributions of cell types within aggregate peripheral blood samples, a uniform procedure was employed. Subsequently, the variations across diverse cell categories among different cohorts were graphically represented.

### Trajectory analysis

The differentiation pathways within the identified cell clusters were examined using the Monocle2 algorithm (16). To isolate the cell clusters of interest, we employed the “subset” function from the Seurat package, followed by the construction of a CellDataSet object with the “newCellDataSet” method in Monocle2, setting the “lowerDetectionLimit” to 0.5. To enhance the quality of the dataset, low-quality cells and genes were filtered out by employing the “detectGenes” and “subset” methods with the “min_expr” threshold set at 0.1. This step occurred after size factor computation and dispersion estimation. Differential gene expression along the determined trajectories was identified using the “differentialGeneTest”. Dimensionality reduction was accomplished through the “reduceDimension” function, leveraging the “DDRTree” approach. For visualization, functions such as “plot cell trajectory”, “plot genes in pseudotime”, and “plot genes branched heatmap” were implemented following cell ordering. Additionally, a CytoTRACE analysis, which is a method for the unsupervised prediction of cells’ relative differentiation states from their single-cell transcriptomes, was conducted (17). This analysis was carried out using the default parameters specified in recommended protocols to augment our understanding of cell trajectory. Visualization of the results was achieved through the “plotCytoGenes” and “plotCytoTRACE” functions.

### Cell communication analysis

The “CellChat” R package (available at https://www.github.com/sqjin/CellChat) (4) facilitated the construction of CellChat objects, with the UMI count matrices pertinent to each subset (Normal and AD) serving as the foundation. The “CellChatDB.human” database was prioritized for ligand-receptor pairings during the analysis. The examination of cellular communication was executed with the preconfigured default settings. Subsequently, to discern the cumulative interaction count and the comparative intensity of these interactions, CellChat objects respectively to each subset were amalgamated via the “mergeCellChat” command. To display the variances in interaction numbers or strengths across different cellular types between Normal and AD groups, both “compareInteractions” and “netVisual_circle” functions were employed. Lastly, the “netVisual_bubble” function allowed for the illustration of the signaling gene expression distribution across the groups.

### WGCNA analysis

WGCNA, a method used for the construction of gene co-expression networks in GSE65682, was facilitated by the WGCNA package in R. The steps for processing were as outlined: initially, genes with missing values were filtered out using the ‘goodSamplesGenes’ function. An optimal soft-thresholding power was then visually selected to ensure a robust network construction. Subsequently, the gene expression data were transformed into an adjacency matrix, and this was further converted into a topological overlap matrix (TOM) to map out genetic interconnections. By examining TOM dissimilarities, genes were clustered using average linkage hierarchical clustering. The clustering dendrogram was dynamically cut to delineate highly correlated modules. The module eigengenes (MEs) served as the representative core of each gene cluster, capturing the module’s overall gene expression profile. The association between MEs and clinical traits was assessed using Pearson correlation to establish their relevance. In conclusion, the focus was on genes within modules that exhibited the strongest correlation to the sphingolipid score for downstream investigation.

### Construction and validation of the risk scoring

To conduct a univariate examination of the intersecting genes to uncover those statistically linked to the patient’s overall survival rate, a significance threshold of P<0.05 was adopted. This analysis was implemented using R, initiating with data preparation which involved importing the gene expression and survival data into R. After ensuring proper data structuring with the ‘survival’ package, univariate Cox proportional hazards regression was employed. This facilitated the identification of genes with significant prognostic value based on their P-values being less than 0.05 through the coxph function.

Subsequent refinement involved leveraging the LASSO (Least Absolute Shrinkage and Selection Operator) Cox regression analysis, carried out using the ‘glmnet’ package. Here, a matrix was constructed from the expression data of the significant genes identified from the univariate analysis, and a corresponding response vector containing survival times and event status was prepared. With the LASSO method being sensitive to the values’ scales, standardization was ensured before model fitting. The model fitting was performed using the cv.glmnet function to identify the optimal parameters via cross-validation, focusing on the lambda value that minimized the cross-validated error (17, 18). Through coef, the best set of genes and their associated risk coefficients, having significant associations with patient outcomes, were selected.

For survival analyses, the log-rank (Mantel-Cox) methodology was operationalized to find the gene group with the most significant prognostic value. This process was facilitated through the survdiff function, which compared survival curves across different gene expression groups, and the gene group achieving the lowest P-value was noted.

Risk scores for each sepsis patient were subsequently calculated from the coefficients derived from the log-rank test. These scores allowed for stratification of patients into high-risk and low-risk groups based on the median value of the risk scores, ensuring clear demarcation between the two cohorts.

Kaplan-Meier plots, generated using the survfit function from the ‘survival’ package, visually represented the survival probabilities over time for both risk groups, providing a clear prognosis evaluation through survival curves. To further scrutinize the predictive model’s performance, ROC (Receiver Operating Characteristic) curves were constructed using the ‘pROC’ package, focusing on the measurement of sensitivity and specificity across varying thresholds.

Finally, the robustness and generalizability of the derived prognostic signature were assessed across four independent datasets. The model’s Area Under the Curve (AUC) values were calculated using roc function from the ‘pROC’ package, serving as a critical validation measure to confirm the model’s consistent performance across different patient cohorts.

### Assessment of the prognostic model

To estimate the 28-day overall survival probabilities, a predictive nomogram was constructed, which includes age, gender, and a composite risk score as separate prognostic determinants. To assess the predictive precision of the nomogram, calibration plots were generated. Additionally, the clinical utility and added value of the nomogram were evaluated through decision curve analysis (DCA), comparing its net benefit to the use of clinical characteristics in isolation.

### Enrichment analysis

Utilizing the “clusterProfiler” R package, as previously specified in the literature (8), we executed enrichment analyses for the KEGG and GO. The scope of the GO biological function covered three domains: BP, MF, and CC. To determine statistical relevance, p-values less than 0.05 were identified as significant.

Furthermore, the Gene Set Variation Analysis (GSVA) was conducted using the ‘GSVA’ R package to elucidate the heterogeneity of biological processes and the activity of various pathways (19). For GSVA, hallmark gene sets from the MSigDB database were selected as the targeted gene sets. The “limma” R package was instrumental in identifying significant differences between biological functions and signaling pathways, with the threshold for statistical significance set to GSVA scores exceeding an absolute t-value of 2.

Additionally, gene set enrichment analysis (GSEA) was conducted to probe the differences in pathway activities, using “clusterProfiler” (20). Pathway activities were ranked according to the Normalized Enrichment Score (NES), with a p-value below 0.05 maintained as the criterion for statistical significance.

Lastly, the activity scores of key disease-related signaling pathways across different cohorts were assessed using the progeny R package, with p-values under 0.05 considered to ascertain statistical significance.

### Assessing the scores of different phenotypes

To discern the distinct phenotypic signatures—namely, those relevant to cholesterol efflux, lysosomal activity, endoplasmic reticulum (ER) stress, angiogenesis, phagocytic function, hypoxic response, acute inflammation, autophagy, and ferroptosis—pertinent gene markers were retrieved from the Molecular Signatures Database (MSigDB). Subsequently, we employed the AUCell algorithm, applying its standard parameters, to calculate phenotype-associated scores across various groups. This process was facilitated by utilizing the irGSEA package.

### Sepsis immunity

The levels of immune cell infiltration were analyzed utilizing the ssGSEA method incorporated within the GSVA softwere (9). In essence, the relative proportions of diverse immune cells were quantified across all samples by leveraging universally recognized gene markers. Subsequently, these algorithms were implemented to ascertain the degree of enrichment or relative quantities for each category of the immune cell. Assessment of the variations in immune cell infiltration across different groups was performed using the Wilcoxon rank-sum test. To depict the extent of immune cell penetration within each AD specimen, divided by algorithm, heatmaps served as a visual aid. Furthermore, the “ESTIMATE” R script played a role in deducing the levels of immune infiltration in patients afflicted with sepsis. Moreover, immune checkpoints consist of an array of molecules such as those involved in antigen presentation, cellular adhesion, co-inhibition, co-stimulation, ligand engagement, and receptor activity—found on immune cells—which modulate the intensity of the immune response. As critical regulators in averting overactive immune responses, we scrutinized and contrasted the expression rates of renowned immune checkpoint genes between the cohorts.

### Establishment and verification of a sepsis rat model with altered PIM1 expression

To investigate the role of PIM1 in sepsis, two cohorts of Sprague-Dawley male rats weighing 250-300g were developed. These animals were raised in a controlled environment with regulated temperature, humidity, and a 12-hour light/dark cycle, and were given unrestricted access to food and water. The animal procedures were approved by the animal ethics committee of Fujian Medical University. The sepsis condition was induced via the well-established cecal ligation and puncture (CLP) technique, which was performed under aseptic conditions and after administering anesthesia (50 mg/kg sodium pentobarbital intraperitoneally). The cecum was ligated, punctured while preserving intestinal continuity, and then returned to the abdomen. Sham-operated rats received all surgical interventions except the CLP procedure. Postoperative care included rehydration through subcutaneous administration of saline. After 24 hours post-operatively, whether CLP or sham, the rats were sedated, and peripheral blood was drawn from the heart into EDTA tubes, subsequently centrifuged, and the samples were preserved for future examination.

For a detailed study on the role of PIM1 in sepsis, a model with diminished PIM1 expression was additionally created via *in vivo* silencing. Adenoviral vectors containing shRNA sequences that specifically target the PIM1 gene in rats (shPIM1) were employed, in comparison with a non-targeting control shRNA sequence (shNC), both of which were procured from RiboBio, located in Guangzhou, China. The experimental group rats were injected via the tail vein with about 30 billion PFU of shPIM1 in 200 μL saline, whereas the control group received an equivalent dosage of shNC. The potency of gene suppression was evaluated on the 14th day following injection through qRT-PCR. Blood RNA isolated with Trizol reagent was subjected to qRT-PCR with PIM1-specific primers for quantitative expression analysis. On the day of analysis, sepsis was induced in the genetically altered subjects, and blood samples were taken using the same collection and preservation method as before for further analysis.

### RT-qPCR

Peripheral blood samples were used to isolate total RNA employing Trizol reagent (Life Technologies, USA). The isolated RNA was subsequently reverse-transcribed to cDNA using the RevertAid First Strand cDNA Synthesis Kit, following the manufacturer’s protocol. Quantitative RT-PCR analyses were performed with the ABI PRISM 7500 system (Applied Biosystems, USA) using the SYBR Premix EX Taq (Takara, Japan) kit. The relative quantification of mRNA expression levels was achieved by normalizing the CT values of the target gene against those of β-actin, with results presented as relative fold changes calculated by the comparative 2-ΔΔCT method.

### Enzyme-linked immunosorbent assay

Peripheral rat blood was collected and the concentrations of cytokines IL-17A, IL-6, TNF-α, and IL-10 were measured using ELISA following protocols supplied by R&D Systems, USA. The blood samples were centrifuged at 2000g for 10 minutes and the supernatants were subsequently harvested for analysis. A reagent diluent was dispensed into each microplate well before the addition of either a blood sample or a standard control. The microplates were sealed and incubated for 2 hours at ambient temperature. After incubation, the contents of the wells were discarded, and the wells were washed thrice. A conjugate reagent (100 µL) was then added to each well, followed by a secondary incubation at room temperature for 2 hours. A subsequent aspiration and washing step was performed before the addition of 100 µL of substrate solution to each well. After a 20-minute incubation, the enzymatic reaction was halted with 50 µL of stop solution. Optical densities at 450 nm were immediately recorded using a spectrophotometer. Cytokine concentrations were quantified against established standard curves.

### Statistical analysis

The R platform was utilized for the management and calculation of our dataset and statistical figures. The survival comparison across the two cohorts was conducted by analyzing Kaplan-Meier plots in conjunction with a log-rank assessment. The ‘ggsurvplot’ package in R facilitated the construction of all survival plots. Prognostic determinants were assessed through univariate Cox regression. The Lasso technique within Cox regression was applied to pinpoint factors with a more substantial impact on the outcomes. Visualization of data points was conducted using ggplot2 in R, while overall survival computations were performed with the survival package. To deduce the association between a pair of continuous variables, Spearman’s rank correlation was executed. The disparities in continuous data between the cohorts were probed via either the Wilcoxon rank-sum test or the two-tailed t-test. Chi-square assessments were put to use for the analysis of categorical variable differences between groups. All statistical evaluations were conducted within the R environment. A P-value below 0.05 was regarded as a threshold for statistical significance.

## Result

### scRNA-seq analysis of GSE175453

**Figure 1:**
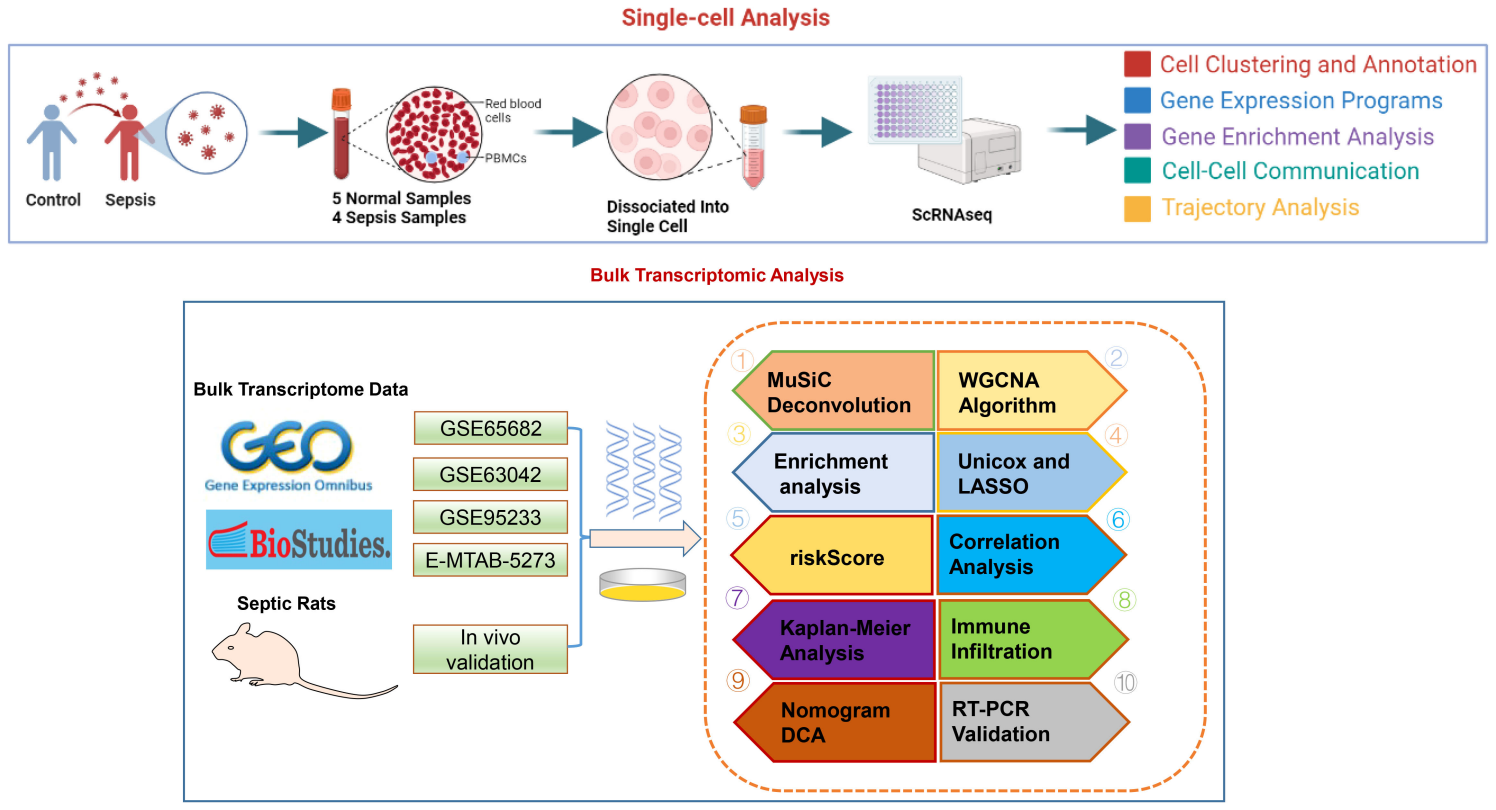
The study flow chart.

**Figure 2:**
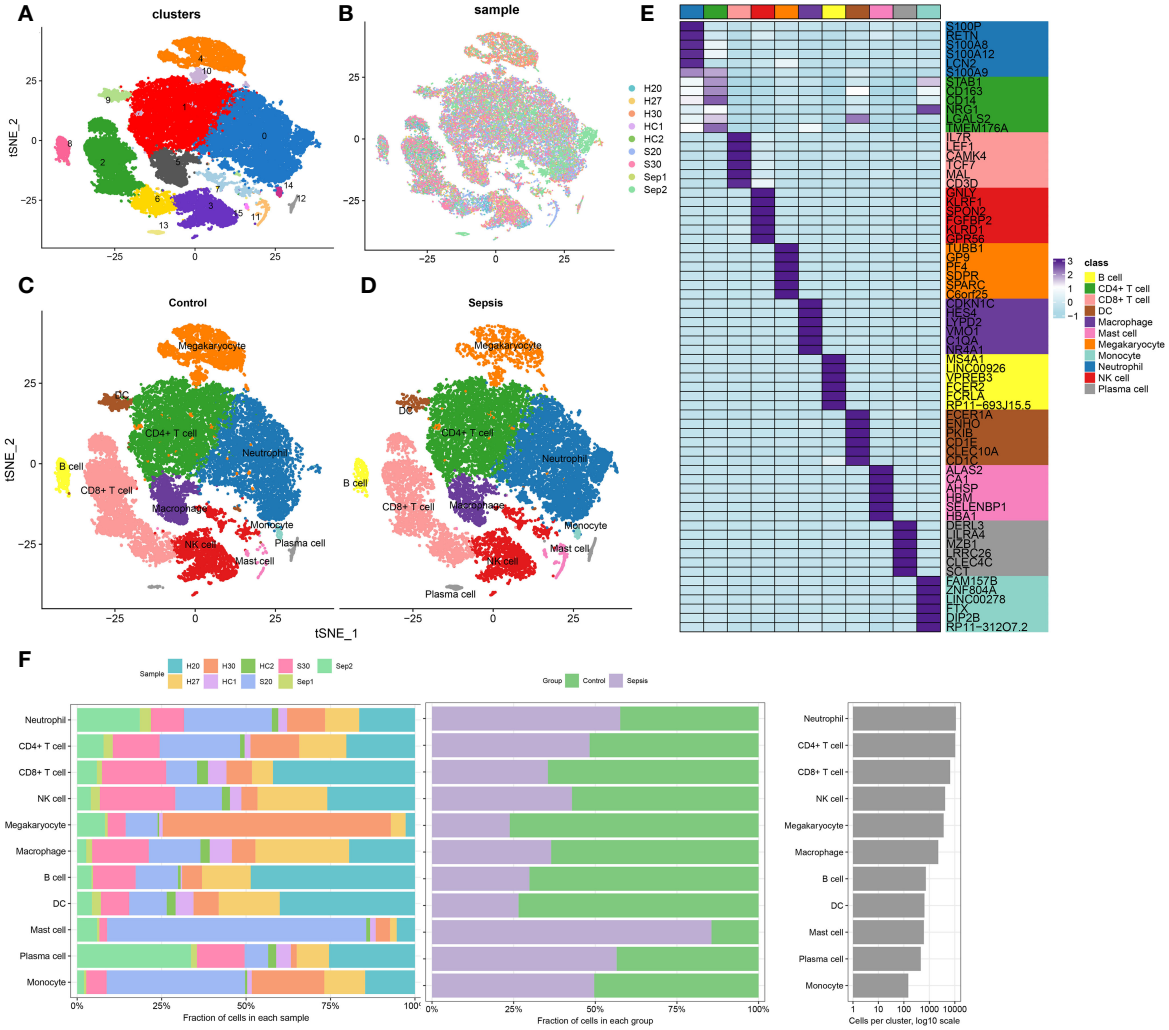
scRNA-seq cell annotation. **(A)** The UMAP plot display distribution of the cell clusters of GSE175453. **(B)**The UMAP plot display distribution of the cell clusters of 5 Healthy control and 4 late septic patients. **(C)** The UMAP plot display distribution of the cell types of Healthy control. **(D)** The UMAP plot display distribution of the cell types of late sepsis patients. **(E)** A heatmap displayed the distribution of the top 6 differentially expressed genes specific to different cell subtypes. **(F)** Cell type fractions of 5 Healthy control and 4 late septic patients.

The methodology of this study was delineated in a flowchart (**Figure 1**). scRNA-seq analysis was employed to extensively characterize the immune cell landscape within the dataset GSE175453. After quality control, a total of 40,584 high-quality cells were obtained, with 22,196 cells derived from healthy controls and 18,388 from sepsis samples, all deemed appropriate for further analysis. **Figure 2A** illustrates the distribution of cell clusters in GSE175453, revealing 15 clusters and 11 immune cell types, categorized as follows: Neutrophils (CD3FR-marked), CD4^+ T cells (CD4-marked), CD8^+ T cells (CD8B-marked), Natural Killer (NK) cells (GNLY-marked), megakaryocytes (TUBB1-marked), macrophages (C1QA-marked), B cells (MS4A1-marked), dendritic cells (DCs; FCER1A-marked), mast cells (CPA3-marked), plasma cells (DERL3-marked), and monocytes (VCAN-marked) with respective cell counts shown in **Supplementary Figure S1**. The distribution of these cell clusters within each sample, control group, and sepsis group is depicted in **Figures 2B-D**, respectively. Furthermore, **Figure 2E** illustrates the six most characteristic genes for each cell type, and **Figure 2F** depicts the proportional representation of each cell type across all samples in dataset GSE175453.

### Evaluation of metabolic activity at single-cell resolution

**Figure 3:**
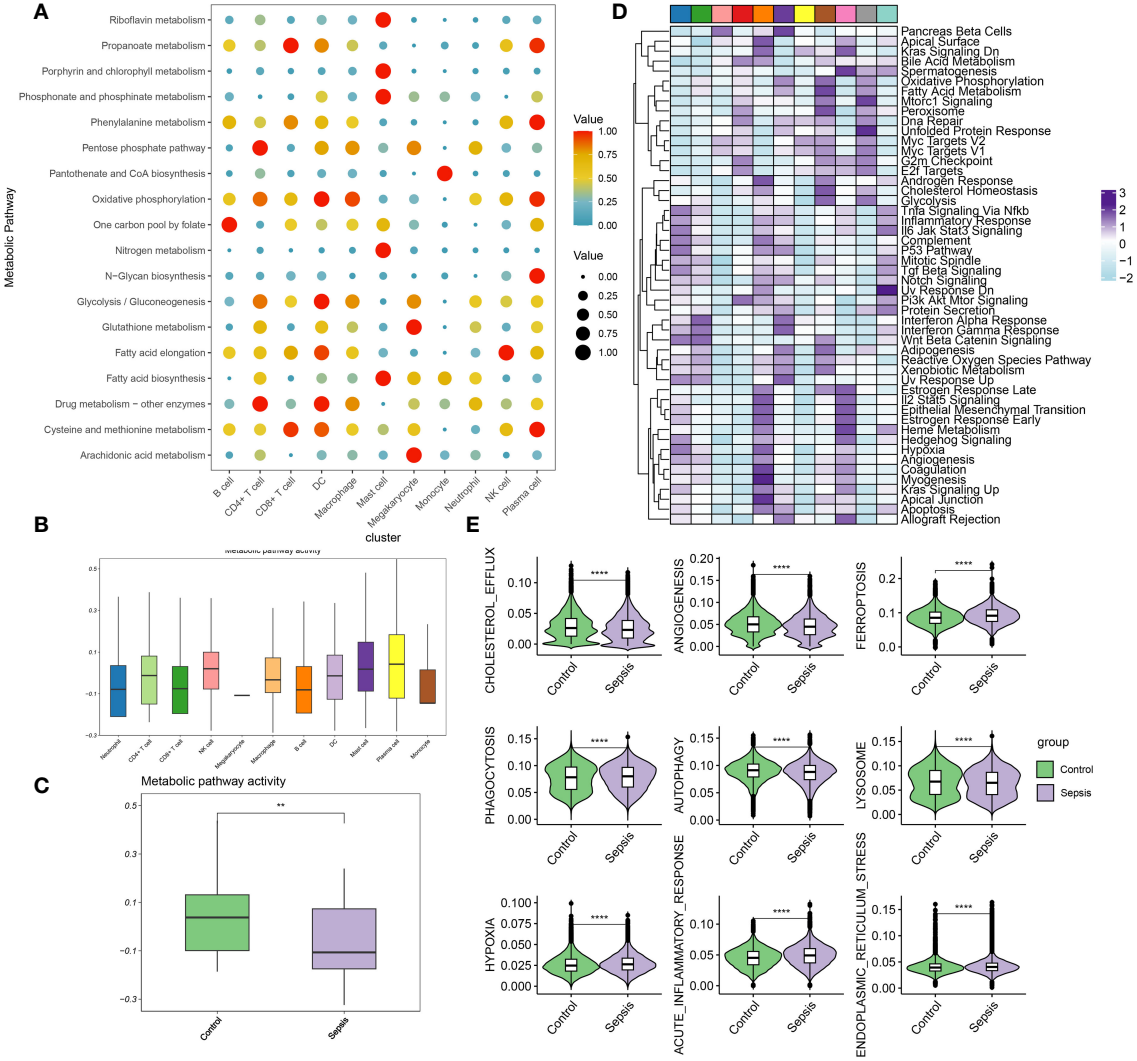
Evaluation of metabolic activity at single-cell resolution **(A)** Dot plots showing differentially metabolic pathways among the global cell subtypes. **(B)** Boxplot showing the metabolic pathway activity among the global cell subtypes. **(C)** Boxplot showing the metabolic pathway activity between control and sepsis group. **(D)** Heatmap showing the differentially enriched pathways among the global cell subtypes. **(E)** Boxplots showing phenotypic scores (cholesterol efflux, lysosome, endoplasmic reticulum stress, angiogenesis, phagocytosis, hypoxia, acute inflammatory response, autophagy, and ferroptosis) between control and sepsis groups. **p < 0.01; ****, *P* < 0.0001.

In this section, the metabolic activities of individual cells in transcriptomic dataset GSE175453 are analyzed. Diverse cell types exhibited enrichment in distinct metabolic pathways, reflecting their unique metabolic roles in the context of sepsis. To summarize, B cells are associated with the one-carbon pool by folate metabolism, CD4+ T cells with drug metabolism involving other enzymes and the pentose phosphate pathway, and CD8+ T cells with propanoate metabolism, as well as cysteine and methionine metabolism. Dendritic cells (DC) were linked to oxidative phosphorylation, glycolysis/gluconeogenesis, drug metabolism involving other enzymes, and cysteine and methionine metabolism. Macrophages were noted for oxidative phosphorylation, while mast cells were involved in riboflavin metabolism, porphyrin, and chlorophyll metabolism, phosphonate and phosphinate metabolism, nitrogen metabolism, and fatty acid biosynthesis. Megakaryocytes were related to glutathione metabolism and arachidonic acid metabolism, monocytes to pantothenate and CoA biosynthesis, neutrophils to the pentose phosphate pathway, natural killer (NK) cells to fatty acid elongation, and plasma cells to propanoate metabolism, phenylalanine metabolism, oxidative phosphorylation, N-Glycan biosynthesis, and cysteine and methionine metabolism (**Figure 3A**). The metabolic pathway activity for each cell type is presented in **Figure 3B**. Neutrophils, CD8+ T cells, B cells, and monocytes demonstrated relatively low metabolic activity, whereas NK cells, plasma cells, and mast cells exhibited higher activity. Notably, compared to the control group, the metabolic activity in the immune cells from the sepsis group was significantly reduced (**Figure 3C**). Moreover, the differentially enriched pathways among the global cell subtypes are depicted in **Figure 3D**. Additionally, variations in classical phenotypes between the control and sepsis groups were analyzed, revealing that phenotypes such as cholesterol efflux, angiogenesis, phagocytosis, autophagy, and lysosome activity were more pronounced in the control group, whereas hypoxia, acute inflammatory response, and endoplasmic reticulum stress were predominantly observed in the sepsis group (**Figure 3E**). In conclusion, the findings indicated that metabolic activity is suppressed during sepsis. Among the cell types studied, neutrophils exhibit the lowest metabolic activity, suggesting that neutrophil function may critically regulate metabolic processes in the context of sepsis.

### Intercellular communication analysis of neutrophils in sepsis

**Figure 4:**
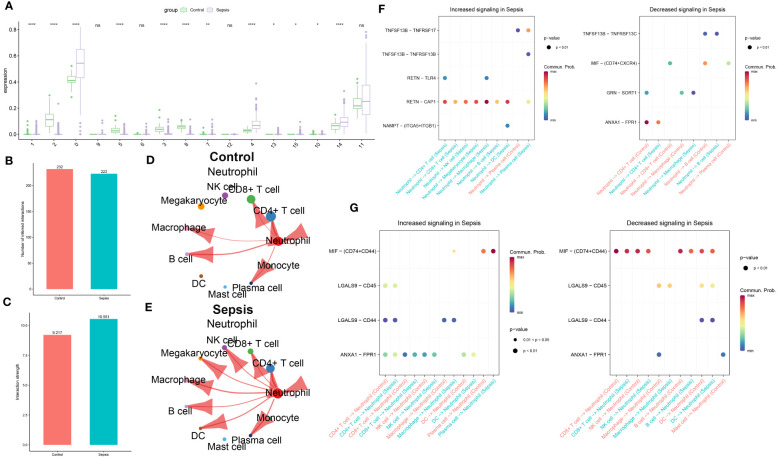
Intercellular communication analysis of Neutrophil in sepsis. **(A)** The distribution of cellular subpopulations within the single-cell cluster in the bulk transcriptome dataset GSE65682. The bar graph illustrates the numbers of inferred interactions **(B)** and interaction strength **(C)** between neutrophils and other cells in the control and sepsis groups in GSE175453. **(D)** The strength of interaction between Neutrophil and other cells in the control group in GSE175453. **(E)** The strength of interaction between Neutrophil and other cells in the sepsis group in GSE175453. **(F)** The neutrophil functions as a ligand that mediates intercellular communication in sepsis. **(G)** Neutrophils functions as a receptor in the pathogenic intercellular communication in sepsis.

The distribution of cell subgroups in the bulk transcriptome dataset GSE65682 was estimated within the single-cell set using the MuSiC algorithm. Notably, it was observed that neutrophils were most prominently enriched in the sepsis group, correlating significantly with metabolic activity (**Figure 4A**). Therefore, in subsequent analyses, Neutrophil was separately extracted for further analysis. In the subsequent analysis, cellular interactions between neutrophils and other cell types were investigated in both control and sepsis groups. As illustrated in **Figure 4B**, a greater number of inferred interactions between neutrophils and other cells were observed in the control group, whereas the interaction strength, depicted in **Figure 4C**, was found to be weaker. In the control group, Neutrophils showed intensive interaction strength and large interaction number with CD4+ T cell, CD8+ T cell, B cell, plasma, macrophage, and Neutrophil (**Figure 4D**). In the sepsis group, Neutrophil displayed strong interaction strength and large interaction number with CD4+ T cell, CD8+ T cell, NK cell, B cell, macrophage, megakaryocyte, DC, mast cell, monocyte, plasma, and Neutrophil (**Figure 4E**). The significant ligand-receptor pairs between neutrophils and other cell types were subsequently further explored. Functions as a ligand, Neutrophil strongly increased the activity of RETN-CAP1 to interact with the majority of receptor cells (CD4+ T cell, CD8+ T cell, NK cell, macrophage, megakaryocyte, B cell, DC, and plasma cell) in the sepsis group and lightly decreased the activity of ANXA1-FPR1 interact with CD4+ T cell (strong), GRN-SORT1 interact with macrophage (light), TNFSF13B-TNFRSF17 interact with plasma cell (light). While Neutrophil only up-regulated TNFSF13B-TNFRSF17 as ligands to interact with plasma cell in control group and decreased the activity of ANXA1-FPR1 interact with CD4+ T cell (strong), MIF-(CD74+CXCR4) interact with CD8+ T cell and plasma cell (light), GRN-SORT1 interact with macrophage (light), MIF-(CD74+CD44) interact with B cell (strong) (**Figure 4F**). Nevertheless, while acting as a receptor, Neutrophils connected with plasma cells by up-regulating MIF-(CD74+CD44) (strong) in both sepsis and control group, but connected with CD8+ T cell, NK cell, B cell, and DC by down-regulating MIF-(CD74+CD44) in both sepsis and control group (**Figure 4G**). In this section, intensive communication between neutrophils and other cell types, particularly within the sepsis group, was observed.

### The development trajectory of neutrophils from control and sepsis samples

**Figure 5:**
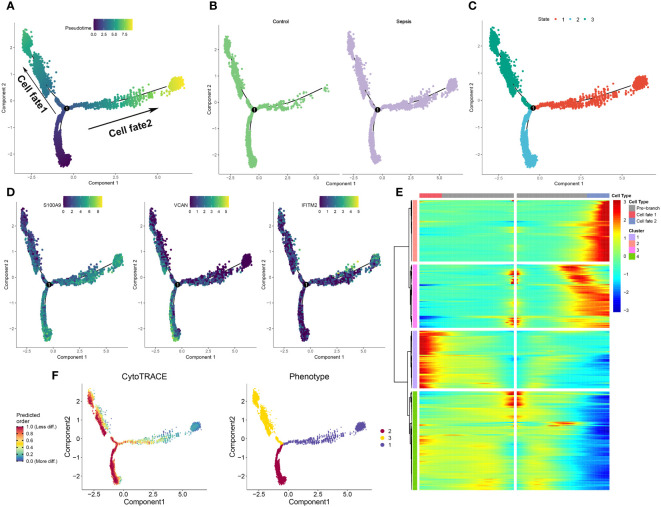
Development trajectory of neutrophils from control and sepsis samples. **(A-C)** The developmental trajectory of neutrophils, colored-coded by the pseudotime **(A)**, group **(B)**, and states **(C)**. **(D)** Representative gene expression in neutrophils during sepsis initiation and progression. Intensity of color indicates normalized gene expression. **(E)**Heatmap showing different blocks of DEGs in each cell fate along the pseudotime of sepsis initiation and progression, colored by cell fates. **(F)** Development trajectory of neutrophils colored by the CytoTRACE scores and Phenotype.

To further elucidate the dynamics of the immune response, a pseudoprime developmental trajectory analysis was conducted on neutrophils, with the objective of fitting the most optimal trajectory curve of cellular development or differentiation in sepsis. This analysis inferred the lineage structure of neutrophils within the atherosclerotic plaque milieu based on the developmental trajectory. As time advanced, the pseudotime analysis delineated the principal evolutionary trajectory of neutrophils, which bifurcated into two unique cellular fates (**Figure 5A**). Subsequently, the developmental trajectories of neutrophils were segregated into control and sepsis groups. Predominantly, neutrophils from the control group were clustered within cellular fate 1, whereas those from the sepsis group were sparsely distributed between both cellular fate 1 and 2 (**Figure 5B**). Neutrophils from the sepsis group were classified into three distinct differentiation states (**Figure 5C**). Furthermore, the differential expression of specific genes (S100A9, VCAN, and IFITM2) was validated within the sepsis trajectory. Of these, S100A9 showed high expression in state 3, with VCAN being chiefly expressed in states 1 and 3, and IFITM2 uniformly present in all three states (**Figure 5D**). The trajectories of lineage-dependent gene expression patterns, accompanying cellular transformations, were further visualized in **Figure 5E**. CytoTRACE predictions suggested neutrophils in states 2 and 3 possess a higher potential for differentiation in sepsis, contrasting with those in state 1 who showed minimal potential (**Figure 5F**). Finally, the phenotypes present within the three cellular states were illustrated, with state 3 incorporating the widest spectrum of phenotypes and state 2 encompassing the narrowest (**Figure 5F**).

### Identification of characteristic genes

**Figure 6:**
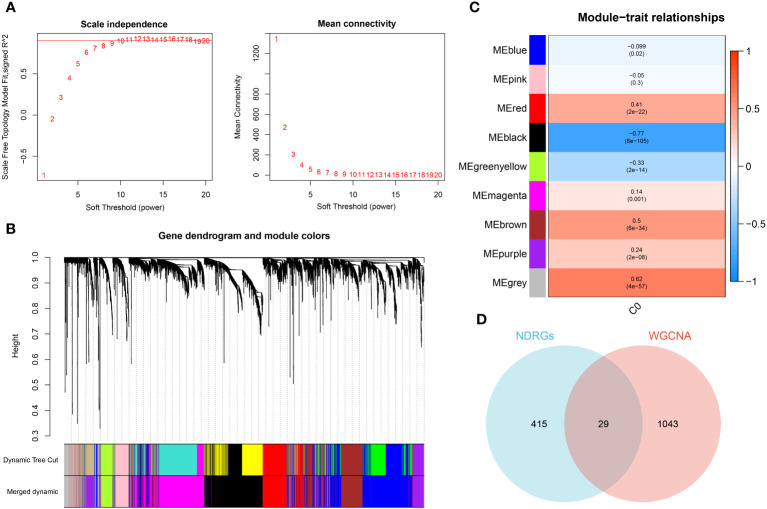
Identification of characteristic genes. **(A)** Ideal soft threshold for adjacency computation of WGCNA. **(B)** Dendrogram of co-expression module clustering. **(C)** The WGCNA analysis investigated the modules of with the most remarkable correlation to neutrophils. **(D)** Interaction of characteristic genes screened from WGCNA and development trajectory analysis of neutrophils.

The WGCNA algorithm was employed to construct a gene co-expression network for GSE65682. By using an optimal soft-thresholding power (β) of 9, a hierarchical clustering algorithm was implemented on the sample data, leading to the identification of nine unique gene co-expression modules, each differentiated by color, in the clustering dendrogram (**Figures 6A, B**). Interestingly, the black module showed the most significant correlation (R=-0.77) with neutrophils, yielding a total of 1,089 genes for further scrutiny (**Figure 6C**). The development trajectory analysis of neutrophils provided 444 neutrophil Differentiation-Related Genes (NDRGs). Following this, an intersection of data from WGCNA and the trajectory analysis resulted in the recognition of 29 characteristic genes, as portrayed in **Figure 6D**.

In the next step, enrichment analyses were carried out to illuminate the potential biological functions of these 29 genes. GO analysis revealed that these genes have a wide-ranging involvement in BP, such as the metabolic process of porphyrin-containing compounds and heme. In terms of CC, the genes could be found in ubiquitin ligase complex, cullin-RING ubiquitin ligase complex, and basal plasma membrane, among others. With regards to MF, these genes were involved in activities such as ubiquitin-protein transferase and ubiquitin-like protein transferase (**Supplementary Figure S2A**). Additionally, the KEGG analysis uncovered substantial enrichment in areas such as bacterial and viral infections, and metabolisms of substances inside and outside cells (**Supplementary Figure S2B**).

### Construction of neutrophils related riskScore system in sepsis

**Figure 7:**
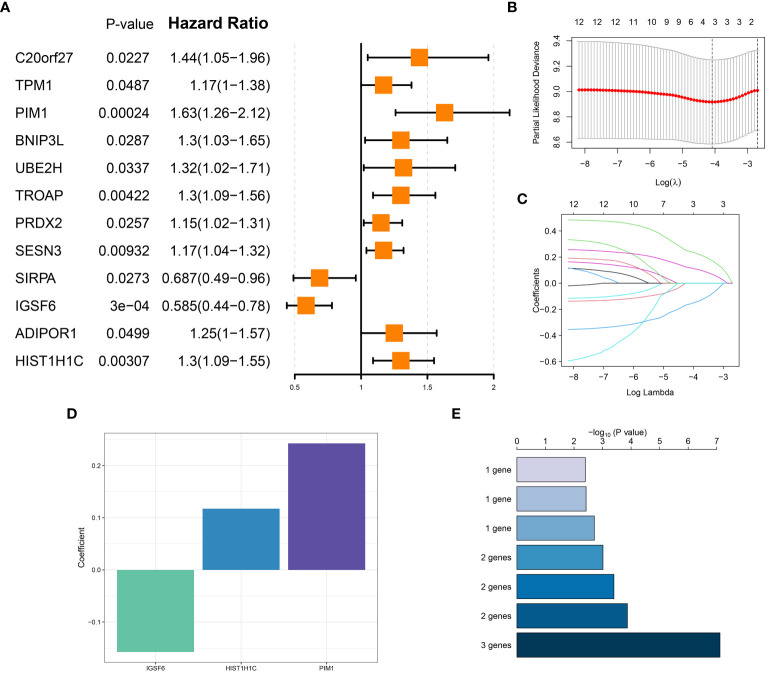
Construction of neutrophils related riskScore system in sepsis. **(A)** Univariate cox analysis on the intersection genes. **(B)** Tuning feature selection in the LASSO model. **(C)** LASSO coefficient profiles of the DDR-related characteristic genes. **(D)** The specific coefficient value of the 3 Genes associated with GM identified by the optimal lambda value. **(E)** Kaplan-Meier analysis of gene combinations, the top 7 signatures were ranked and the signature comprising four genes was selected due to its relatively large negative logarithm (-log10) of the p-value combined with a minimal gene count.

The univariate Cox proportional hazards analysis was performed on 29 NDRGs, revealing 12 genes that demonstrated a statistically significant association with the overall survival of patients in the bulk sepsis transcriptome data GSE65682 (represented as a univariate analysis hazard ratio [HR]) (**Figure 7A**). This was followed by the least absolute shrinkage and selection operator (LASSO) Cox regression analysis and the log-rank (Mantel-Cox) tests to refine the identification of survival-associated genes (**Figures 7B–E**). The analysis culminated in the identification of three hallmark genes (IGSF6, HIST1H1C, and PIM1), based on which the neutrophil-related riskScore model was created. The riskScore calculation is (-0.3196490 × IGSF6) + (0.1483832 × HIST1H1C) + (0.3325431 × PIM1). Patients from the bulk sepsis transcriptome data were divided into high- and low-risk categories using the median riskScore.

### The evaluation of the riskScore system

**Figure 8:**
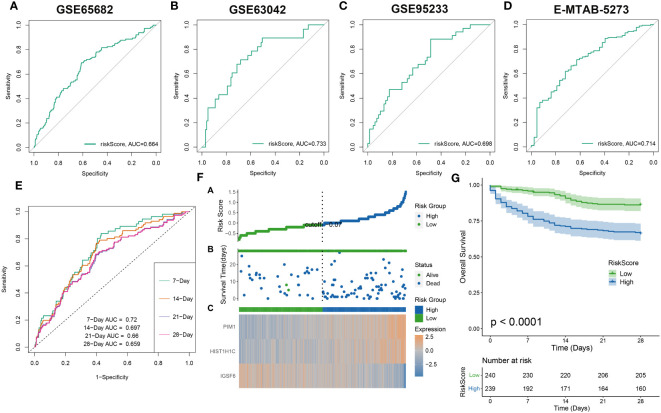
The evaluation of the RiskScore system. The ROC curve was used to evaluate the performance of the riskScore model in the GSE65682 **(A)**, GSE63602 **(B)**, GSE95233 **(C)**, E-MTAB-5273 **(D)**, and the combination dateset **(E)**. **(F)** The distribution of the riskscore, patients’ survival status as well as gene expression signature in the combination dateset. **(G)** Overall survival situation between the low- and high-risk group.

The effectiveness of the riskScore-based prognosis predictive model was evaluated using survival analysis, exhibiting consistency across all assessments. The prediction accuracy of riskScore reflected robustly in the four datasets: GSE65682, GSE63042, GSE95233, and E-MTAB-5273) with AUC values for 28-day mortality exceeding 0.65 (**Figures 8A–D**). This high accuracy continued to prevail in combined dataset evaluations for 7, 14, 21, and 28-day mortality, wherein AUC values all surpassed 0.65 (**Figure 8E**). Further division of sepsis samples into two risk categories, high-risk and low-risk, revealed trends of reduced mean survival periods in high-risk patients, often succumbing in the early illness phase. Low-risk patients revealed a consistent increase of IGSF6 expression as opposed to their high-risk counterparts who showed increased expressions of PIM1 and HIST1H1C (**Figure 8F**). Interestingly, survival analysis reiterated the enhanced survival probabilities for low-risk patients compared to high-risk patients (**Figure 8G**).

**Figure 9:**
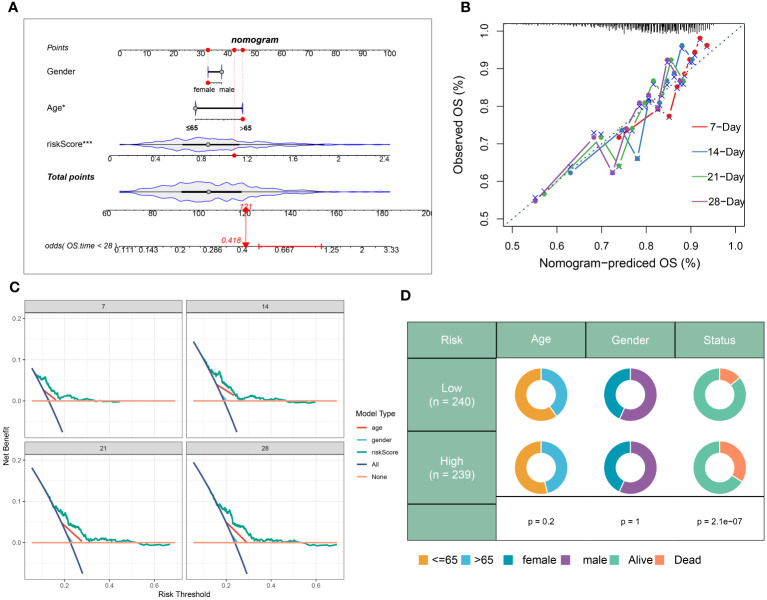
Construction and validation of a prognostic prediction model based on the riskScore. **(A)** Construction of a nomogram based on riskScore and clinical characteristics in the combination dateset. **(B)** Correction of the characteristic curve based on riskscore and pathological characteristic. **(C)** DCA indicating the clinical benefit of the nomogram. **(D)** The distribution of clinical features and survival status in the low- and high-risk groups.

In addition, a prognostic nomogram for sepsis was developed, integrating demographic variables such as age and sex, based on the neutrophils-related risk score. Each predictor in the nomogram warranted a particular score, with the total score across all predictors designating a cumulative score reflecting the likelihood of a negative outcome in sepsis. This cumulative score was visibly represented in **Figure 9A**. The calibration plot verifies the predictive accuracy of the nomogram as shown in **Figure 9B**. The clinical applicability of our nomogram, standing on the calculated risk score, was further substantiated by DCA (**Figure 9C**). Moreover, a schematic representation of the demographic distribution by age, sex, and survival statuses, categorized into two risk groups, has been provided. No significant variation in the age and gender distribution across cohorts was brought to light by this analysis (**Figure 9D**).

**Figure 10:**
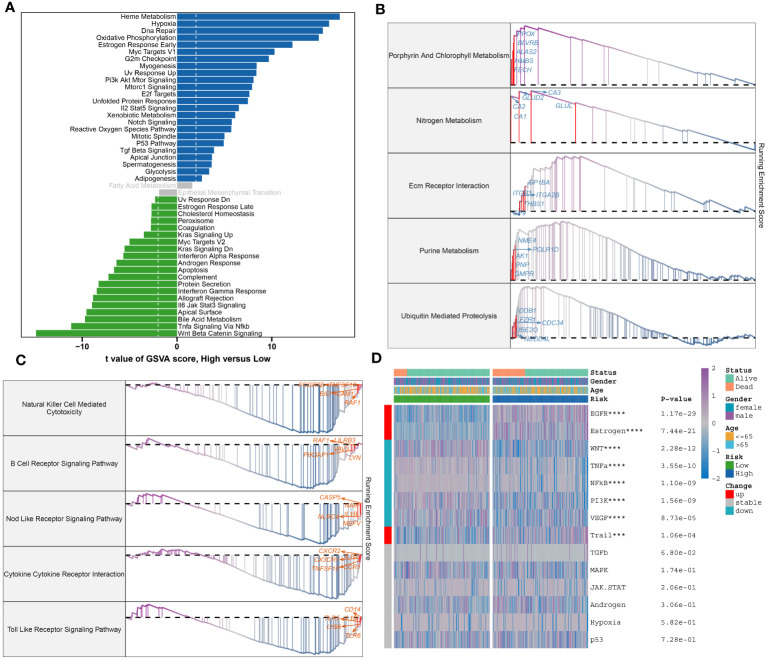
Molecular characteristic and functional annotation of the neutrophils-related riskScore model in sepsis. **(A)** The GSVA identified significant differences in biological functions between the high- and low-risk groups. Positive values indicate that the biological function is enriched in the high-risk group, while negative values indicate that the biological function is enriched in the low-risk group. **(B)**Top five up-regulated pathways in the high-risk group. **(C)**Top five pathways down-regulated in the high-risk group. **(D)** Heatmap displaying the difference of pathogenic pathways in sepsis patients at low and high risk. Age, gender, and survival status are displayed as patient annotations. ***p < 0.001, ****p < 0.0001.

To further elucidate the neutrophil-associated mechanisms in sepsis, characteristic genes of both high- and low-risk groups in GSE65682 were examined. Upon identification, these said genes were put through enrichment analysis using both GSVA and GSEA methods. Divergent pathway enrichment patterns were observed between the two groups. In the high-risk group, notable enrichment was seen in pathways relating to Metabolic Processes, Cellular Stress Responses, and Cell Cycle. This encompassed pathways such as Heme Metabolism, Hypoxia, Oxidative Phosphorylation, Estrogen Response Early, Pi3k Akt Mtor Signaling, Mtorc1 Signaling, E2f Targets, Unfolded Protein Response, Xenobiotic Metabolism, Notch Signaling, Reactive Oxygen Species Pathway, Mitotic Spindle, and P53 Pathway. Conversely, the low-risk group demonstrated significant involvement in several biological functions critical to immuno-inflammatory responses, namely the Interferon Alpha Response, Androgen Response, Apoptosis, Complement, Protein Secretion, Interferon Gamma Response, Allograft Rejection, Jak-Stat3 Signaling, Bile Acid Metabolism, Tnf-a Signaling Via Nf-kb, and Wnt Beta Catenin Signaling pathways (**Figure 10A**). Proceeding with the investigation, the top 5 up-regulated (Porphyrin And Chlorophyll Metabolism, Nitrogen Metabolism, Nitrogen Metabolism, Purine Metabolism, Ubiquitin Mediated Proteolysis) and top 5 down-regulated pathways (Natural Killer Cell-Mediated Cytotoxicity, B Cell Receptor Signaling Pathway, Nod Like Receptor Signaling Pathway, Cytokine Cytokine Receptor Interaction, Toll-Like Receptor Signaling Pathway) within the high-risk group were further discerned through GSEA (**Figures 10B, C**). Pathogenetic pathway variability was also evident among different-risk sepsis patients. Particularly, high-risk patients demonstrated significant activity in the EGFR, Estrogen, and Trail pathways. On the other hand, low-risk patients showed hyperactivity in the WNT, TNF-α, NF-KB, PI3K, and VEGF pathways compared to their high-risk counterparts (**Figure 10D**).

### Immunological features of sepsis patients at low and high risk

**Figure 11:**
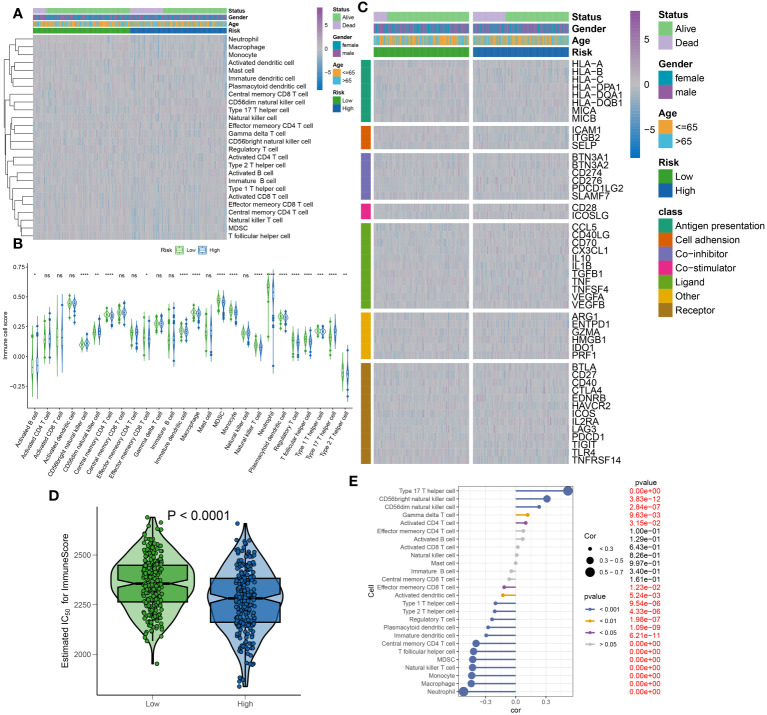
Immunological features of sepsis patients at low and high risk. **(A)** The heatmap showing the degree of infiltration of 26 immune cell subtypes in high- and low-risk groups. **(B)** Differences in immune cell scores between high- and low-risk groups. **(C)** Heatmap depicting the differences in immune-modulators and patients’ survival status between high- and low-risk groups. **(D)** A comparison of the immuneScore between high- and low-risk groups. **(E)** The interaction between riskScore and 26 immune cell subtypes.

To elucidate the infiltration of immune cells in patients with sepsis categorized into high- and low-risk groups, each further classified by stable or unstable clinical statuses, a comparative analysis of 26 immune cell subtypes was initially performed. This was executed through the calculation of the 26 immune cell scores using the ssGSEA algorithm (**Figure 11A**). Generally, the low-risk group displayed higher levels of immune cell infiltration compared to the high-risk group, aligning with previous results that exhibited higher immune cell scores in the majority of immune cell types within the low-risk group (**Figure 11B**). Moreover, the variations in immune modulators between the high- and low-risk groups were evaluated, based on different statuses, genders, and ages, as an attempt to further clarify the immune characteristics of sepsis patients (**Figure 11C**). In summary, immune genes associated with antigen presentation (HLA-DQA), cell adhesion (SELP), co-inhibitor (CD276 and PDCD1LG2), co-stimulator (ICOSLG), ligand (CCL5, CD40LG, CD70, CX3CL1, and VEGFB), receptor (CD27, EDNRB, IL2RA, LAG3, and PDCD1), among others (PRF1), were visibly elevated in the high-risk group. However, low-risk samples illustrated substantial expression of antigen presentation (HLA-A, HLA-B, HLA-C, MICA, and MICB), cell adhesion (ICAM1 and ITGB2), co-inhibitor (CD274 and SLAMF7), ligand IL1B, TGFB, and TNF), receptor (CD40, HAVCR2, TIGIT, TLR4, and TNFRSF14), among others (ENTPD1) (**Supplementary Figures S3A–G**). Furthermore, a comparative analysis of immune scores from each risk group was carried out, yielding a comprehensive review of immunological attributes. Patients in the low-risk group achieved higher immune scores compared to those in the high-risk group (**Figure 11D**). Additionally, a correlation analysis indicated that elevated risk scores negatively affected the entirety of immune cell types and demonstrated higher immune infiltration levels (**Figure 11E**). Based on the findings of our study, a relative decrease in the activity of immune cells, immune responses, and immune-related pathways was observed in high-risk sepsis patients, indicating a symptomatic immune suppression during sepsis.

### Validation of hallmark genes in a rat model of sepsis

**Figure 12:**
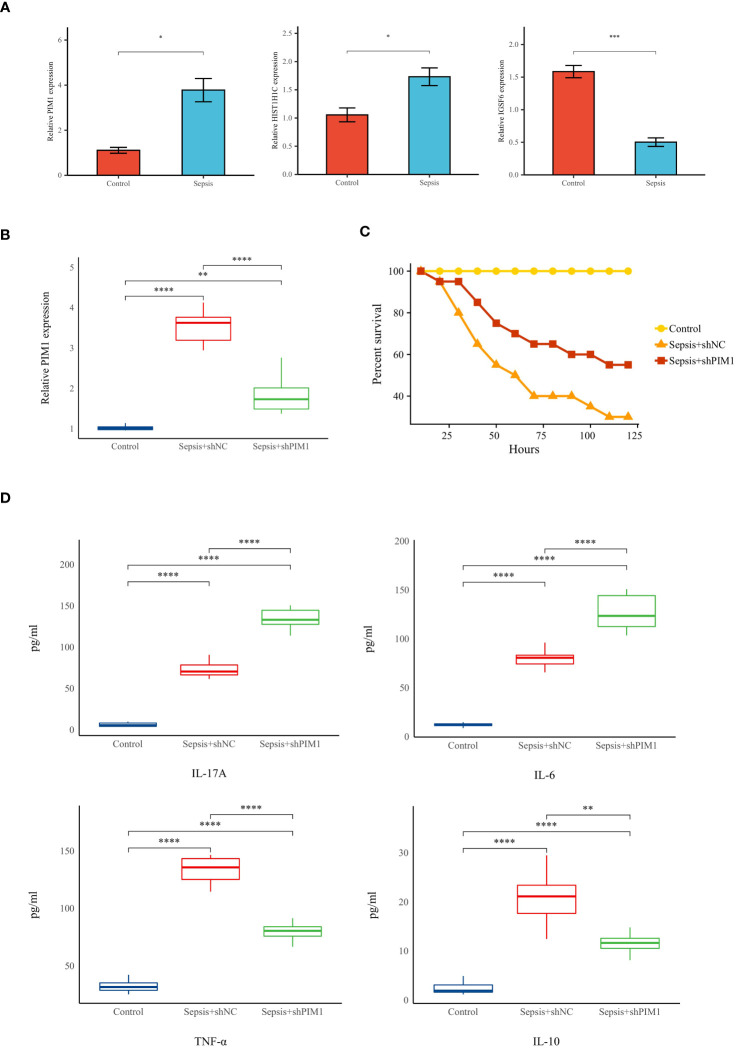
Validation of hallmark genes in a rat model of sepsis **(A)** Relative expression levels of hallmark genes in Control and Sepsis groups (n=5 in each group). **(B)**Relative expression levels of PIMI in Control, Sepsis+shNC, and sepsis+shPIMI group (n=8 in each group). **(C)** Survival status of rats in each group (n=10 in each group). **(D)**The level of pro-inflammatory cytokines (IL-17A, TNF-a, and IL-6) and anti-inflammatory cytokines (IL-10) in the peripheral blood of rats in each group (n=8 in each group). *p < 0.05, **p < 0.01, ***p < 0.001, ****p < 0.001.

To substantiate the involvement of signature genes in the development of a neutrophil-related risk score model for sepsis, *in vivo* validation experiments were conducted. Initially, a sepsis model was established in rats, followed by an analysis of gene expression in their peripheral blood via reverse transcription-quantitative polymerase chain reaction (RT-qPCR). Among the genes studied, PIM1, HIST1H1C, and IGSF6 demonstrated a marked upregulation in the septic rats (see **Figure 12A**). PIM1 was selected for in-depth validation due to its significant contribution to the risk score model. Subsequent RT-qPCR evaluations revealed that PIM1 expression in the Sepsis+shPIMI group was reduced to nearly one-third compared with the Sepsis+shNC group, confirming effective gene silencing within our framework (refer to **Figure 12B**). Survival analyses further showed that rats subjected to PIM1 knockdown presented enhanced survival rates relative to the Sepsis+shNC cohorts (as indicated in **Figure 12C**). Additionally, the levels of pro-inflammatory cytokines, such as IL-17A, IL-6, and TNF-α, were notably elevated in the Sepsis+shPIMI group, whereas the anti-inflammatory cytokine IL-10 was reduced (depicted in **Figure 12D**). These findings identify PIM1 as a potential pivotal modulator of immune and inflammatory responses during sepsis. The section that follows will provide additional evidence of the critical role played by PIM1 in sepsis, suggesting its potential involvement in the immunosuppressive mechanisms of the disease.

## Discussion

During sepsis, metabolic changes in the patient’s body not only contribute to early inflammation and organ damage but also play significant roles in immune tolerance and immune exhaustion (11). Sepsis is marked by significant metabolic dysregulation across various pathways, including carbohydrate, amino acid, and fat metabolism (21). Leukocytes from patients with severe sepsis exhibited profound defects in cellular energy metabolism, which were correlated with a diminished capacity to respond to secondary stimulation (11). In the pathogenesis and progression of sepsis, further research is needed to elucidate the intricate mechanisms and heterogeneity of various immune cells influencing metabolism. Such research is crucial for establishing a robust theoretical foundation to advance personalized clinical interventions for sepsis.

In this study, scRNA-seq was utilized to delineate the immune landscape in both healthy controls and patients with late-stage sepsis. We identified distinct distributions of immune cells and metabolic activity profiles between the groups. Remarkably, immune cells from septic patients exhibited a broadly reduced metabolic activity compared to those from healthy controls, likely due to metabolic exhaustion related to the severe inflammatory response during sepsis. The observed hypometabolic state in sepsis may serve as a protective mechanism against excessive inflammation or energy depletion, or it may indicate a dysfunctional immune response. Among all immune cell types, neutrophils played a pivotal role in the immune response and metabolic activity during sepsis. Neutrophils were the most abundantly expressed and demonstrated the lowest metabolic pathway activity in the septic group, while also interacting significantly with other immune cells. During sepsis, neutrophils exhibited enhanced longevity and reduced migratory capabilities, leading to their retention within the vascular system. Consequently, this promotes excessive vascular inflammation through the secretion of cytokines, reactive oxygen species, and neutrophil extracellular traps (22). As the first responders of the innate immune defense against infection, neutrophils utilize traditional mechanisms such as phagocytosis alongside the release of inflammatory cytokines and ROS. In addition to these mechanisms, activated neutrophils release web-like structures comprising decondensed DNA, histones, myeloperoxidase, and other granular contents, known as neutrophil extracellular traps (NETs), which effectively ensnare bacteria within the bloodstream (23). Although the prevailing response of immune cells in sepsis is to undergo apoptosis, thus promoting an immunosuppressive environment, neutrophils uniquely exhibit delayed apoptosis, further perpetuating the inflammatory response (24). Polymorphonuclear neutrophils possess limited mitochondria and predominantly rely on the comparatively inefficient process of glycolysis for their energy metabolism, which is responsible for generating the bulk of ATP needed for neutrophil functionality (25, 26). During phagocytosis, there is an elevated consumption rate of ATP, and in the context of sepsis, systemic ATP levels can impede neutrophil activation and chemotaxis by disrupting intrinsic purinergic signaling pathways (27). Nevertheless, the specific metabolic traits and immunomodulatory routes of neutrophils during sepsis remain inadequately explored.

This research revealed that intercellular communication demonstrates a complex interaction network between neutrophils and other cell types, potentially underlying the septic process. Ligand-receptor analyses indicated active crosstalk between neutrophils and other cell types during sepsis, highlighting elevated levels of specific proinflammatory mediators in the septic milieu. Moreover, the exploration of developmental trajectories suggested neutrophil plasticity in sepsis, with distinct phenotypes correlating to varying sepsis severity. This plasticity likely represented an adaptive response to the multifaceted stimuli encountered during sepsis. Moreover, 29 neutrophil differentiation-related genes during sepsis were obtained by intersecting feature genes from WGCNA and trajectory analysis. Then we acquired 3 hallmark genes (IGSF6, HIST1H1C, and PIM1) by machine learning approaches, and the neutrophils-related riskScore model consisting of 3 genes was constructed. The immunoglobulin superfamily member IGSF6 was involved in immune regulation and has been linked to the immunological landscape of tumors (28). IGSF6 expression was associated with the infiltration of CD8+ T cells and CD4+ T cells in tumors, indicating an active immune response within the tumor microenvironment (29). Some studies reported the involvement of IGSF6 in the immunoregulation of atherosclerosis and inflammatory bowel disease (30, 31). A recent study identified that IGSF6 regulates ER stress and the inflammatory response in intestinal macrophages. IGSF6 expression is sustained by microbiota and significantly upregulated upon bacterial infection (32). HIST1H1 proteins bind to nucleosomes and facilitate chromatin compaction 1, although their biological functions are poorly understood. According to a recent authoritative study, HIST1H1 was identified as a bona fide tumor suppressor and show that mutations in H1 drive malignant transformation primarily through three-dimensional genome reorganization, which leads to epigenetic reprogramming and derepression of developmentally silenced genes (33). Moloney murine leukemia virus-1 (PIM1) functions as a kinase influenced by cytokine signaling, and its role is particularly pivotal in the context of IFN-γ signaling pathways during infections (34). It appears to act as a sensor detecting a wide array of pathogens that disrupt IFN-γ signaling. PIM1 has a short lifespan within infected cells. PIM1 appears to play a regulatory role in the immune response by controlling the parasiticidal function of GBP1. The regulation of GBP1’s antimicrobial function by PIM1 suggests that this interaction is a part of an IFNγ-induced pathway which provides post-translational control of innate immune defense (35). Additionally, PIM1 also promotes the survival and immunosuppressive function of neutrophils during chronic viral infection, influencing CD8 T cell function and viral control (36).

The neutrophil-related riskScore system reflects vital prognostic information and predicts patient outcomes informatively in sepsis. This aligns with studies advocating for personalized medicine approaches based on immune profiling. Based on the risk scoring, patients were stratified into high-risk and low-risk groups. GSVA and GSEA highlighted marked functional disparities between high- and low-risk sepsis patients. The high-risk group was associated with enrichment of metabolic pathways and stress responses, potentially indicative of the metabolic demands of a sustained inflammatory response. In contrast, the low-risk group demonstrated enrichment in immune functions, suggesting less compromised immune responses. Assessment of immune cell infiltration and immune-modulators unveiled a robust immune phenotype in low-risk patients, likely contributing to the effective response against infection. In contrast, high-risk patients exhibited a subdued immunological profile, which may predispose them to adverse outcomes. In all, in this study, we identify the high-risk group as “immune suppression phenotype”, while the low-risk group is “Immunoactive type”.

Research has established that sepsis-induced immunosuppression stems from dysfunctions in both innate and adaptive immunity. This condition is marked by elevated levels of anti-inflammatory cytokines, the apoptosis of immune cells, T-cell dysfunction, and a heightened presence of immuno-regulatory cells such as regulatory T cells and myeloid-derived suppressor cells (37, 38). Immunological suppression associated with inflammation is a critical determinant in the onset of secondary infections and multiple organ dysfunction syndrome (MODS), which are chief contributors to the adverse prognoses observed in septic patients (39). The results are consistent with the conclusions drawn in this study. It was demonstrated that within the neutrophil-based risk model, patients classified in the high-risk group exhibited significant immunosuppression and metabolic dysregulation. This finding indicates the potential utility of using neutrophil-based metrics as immunological prognostic markers to aid in risk assessment and to identify potential therapeutic targets.

The three genes constituting the risk score were further validated, revealing that their expression levels were significantly higher in the peripheral blood of sepsis-induced rats compared to the control group. Additional experimental validation was subsequently performed on PIM1, the gene with the highest risk coefficient. The inhibition of PIM1 resulted in a significant increase in the inflammatory levels in the peripheral blood of the septic rats. Moreover, the survival rate of the septic rats in the PIM1 knockdown group was higher than that of the septic rats in the control group. These experimental findings were consistent with the conclusions of our previous bioinformatics analysis, confirming that PIM1 may be one of the critical genes involved in the immune suppression observed following the onset of sepsis. These findings highlight the heterogeneity in immune responses among sepsis patients and suggest that a personalized medicine approach, informed by detailed immunophenotyping, could lead to more tailored and effective treatment strategies. Understanding the role of specific immune cells and their metabolic pathways in sepsis may open avenues for the development of immunomodulatory therapies aimed at restoring immune balance rather than just controlling the infection.

Throughout the duration of the research, a series of challenges were encountered, and unexpected discoveries were made: (1) Inter-individual Variability: considerable variability in immune responses and metabolic profiles among sepsis patients was observed. This variability underscores the complexity of sepsis as a syndrome and indicates the potential necessity for personalized therapeutic approaches. (2) Unanticipated dynamic changes in neutrophil subpopulations were revealed by pseudotime analysis. Certain neutrophil states demonstrated unexpected gene expression patterns, suggesting novel roles in the immune response to sepsis. (3) Unexpected interactions between different metabolic pathways, typically studied in isolation, were identified. This cross-talk implies more intricate metabolic reprogramming in immune cells during sepsis than previously recognized.

Several limitations need to be acknowledged in the present study. First, the sample size of sepsis patients included in the scRNA-seq was relatively small, potentially impacting the generalizability of our findings. Future research with larger cohorts is essential to validate the robustness of the identified biomarkers and the risk score model across diverse populations. Second, the current analysis primarily focuses on neutrophils, which, although critical, represent only a fraction of the complex immune response in sepsis. Expanding the scope to include a broader range of immune cells and their interactions will provide a more comprehensive understanding of sepsis immunopathology.

## Conclusion

A comprehensive analysis has provided insights into the complex immune cell interactions and functional pathways associated with metabolic dysregulation in sepsis, with a particular emphasis on neutrophils. Distinct neutrophil subpopulations and their dynamic differentiation patterns have been discovered, contributing to the understanding of immune response variability in sepsis. Key diagnostic biomarkers, including PIM1, HIST1H1C, and IGSF6, have been identified and incorporated into an accurate riskScore model for the prognosis of sepsis. This model stratifies patients into risk categories and provides insights into immune dysfunction associated with poor outcomes. Furthermore, PIM1 has been experimentally validated as a negative regulator of immune-inflammatory response, indicating its therapeutic potential. These findings collectively enhance the understanding of sepsis immunopathology and offer promising directions for prognosis and treatment interventions.

## Data availability statement

The original contributions presented in the study are included in the article/**Supplementary Material**. Further inquiries can be directed to the corresponding author.

## Ethics statement

This study was approved by the Institutional Animal Care and Use Committee of The Second Affiliated Hospital of Fujian Medical University and conducted in accordance with the Guidelines for the Care and Use of Laboratory Animals." (No humans were accessed in this study).

## Author contributions

SJ: Writing – original draft. HZ: Writing – review & editing. QL: Writing – review & editing. JY: Writing – review & editing. RZ: Writing – review & editing. ZX: Writing – review & editing. WS: Writing – review & editing, Writing – original draft.
