# Supplementary material for: Deciphering the immune-metabolic nexus in sepsis: a single-cell sequencing analysis of neutrophil heterogeneity and risk stratification
Source: Front Immunol. 2024 Jul 23;15:1398719. doi: 10.3389/fimmu.2024.1398719 (PMC11300223; doi:10.3389/fimmu.2024.1398719)
Supplement: Supplementary file 1 [file Image_1.pdf]

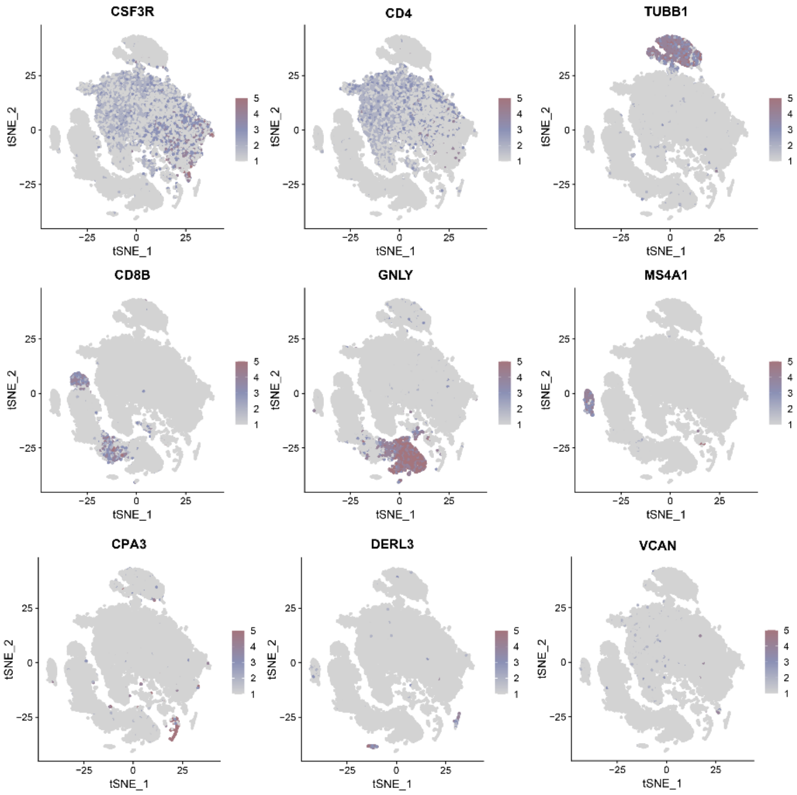

FigureS1 Distinctive marker genes for each cell subtype.

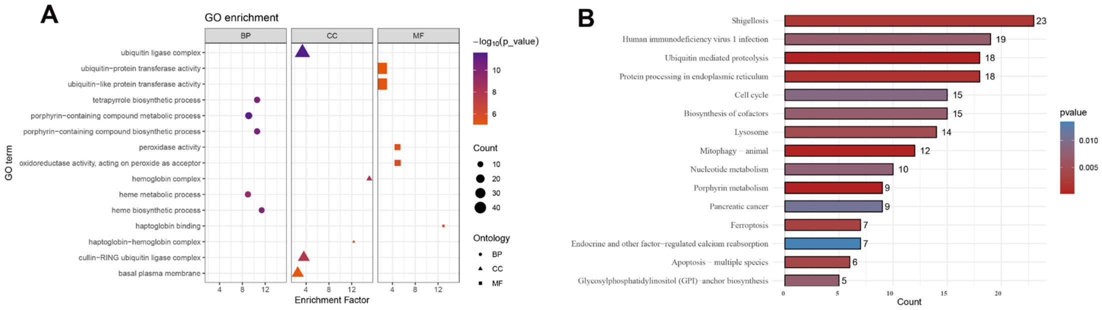

FigureS2 Enrichment analyses of characteristics genes. (A) GO analysis. (G) KEGG analysis .

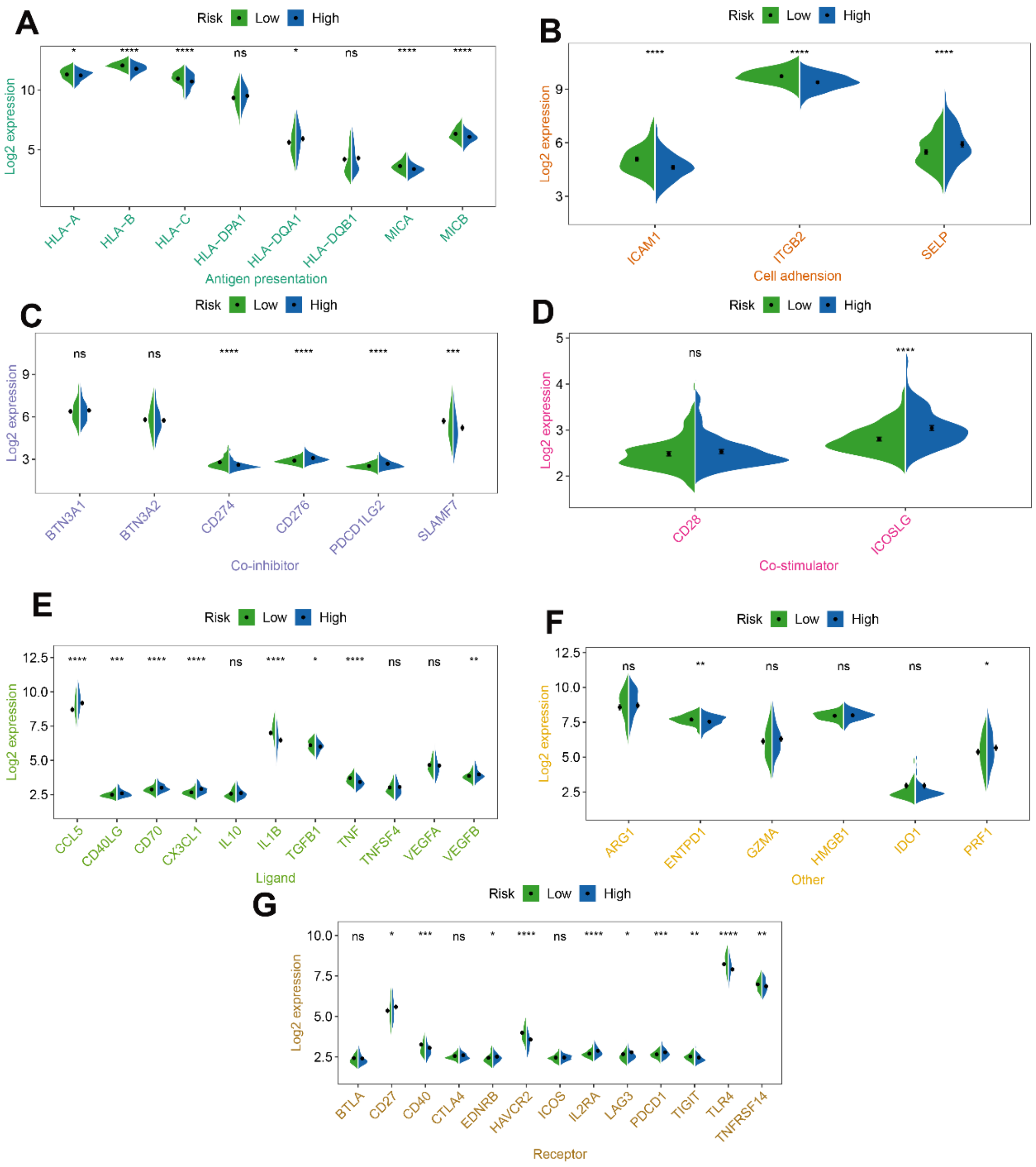

Figure S3 the expression profiles of immunoregulatory subgroup genes in sepsis patients at low and high risk. *$p < 0.05$, **$p < 0.01$, ***$p < 0.001$, ****$p < 0.0001$.
